# Bidirectional Approach with PIPAC and Systemic Chemotherapy for Patients with Synchronous Gastric Cancer Peritoneal Metastases (GCPM)

**DOI:** 10.1245/s10434-023-13572-7

**Published:** 2023-06-03

**Authors:** Francesco Casella, Maria Bencivenga, Giorgio Brancato, Lorena Torroni, Cecilia Ridolfi, Carmelo Puccio, Mariella Alloggio, Francesca Meloni, Daniele Fusario, Daniele Marrelli, Simone Giacopuzzi, Franco Roviello, Giovanni de Manzoni

**Affiliations:** 1 Upper G.I. Surgery Division, University of Verona, Verona, Italy; 2 Department of Diagnostics and Public Health, Unit of Epidemiology and Medical Statistics, University of Verona, Verona, Italy; 3 Department of Medicine, Surgery and Neurosciences, Unit of General Surgery and Surgical Oncology, University of Siena, Siena, Italy

**Keywords:** Gastric cancer, Peritoneal metastases, PIPAC, Bidirectional

## Abstract

**Background:**

This study evaluated the efficacy of pressurized intraperitoneal aerosol chemotherapy (PIPAC) with systemic chemotherapy as a bidirectional approach for gastric cancer (GC) patients with synchronous peritoneal metastases (SPM).

**Methods:**

A retrospective analysis of a prospective PIPAC database was queried for patients who underwent a bidirectional approach between October 2019 and April 2022 at two high-volume GC surgery units in Italy (Verona and Siena). Surgical and oncological outcomes were analyzed.

**Results:**

Between October 2019 and April 2022, 74 PIPAC procedures in 42 consecutive patients with Eastern Cooperative Oncology Group performance status ≤2 were performed—32 patients treated in Verona and 10 in Siena. Twenty-seven patients (64%) were female and median age at first PIPAC was 60.5 years (I–III quartiles: 49–68 years). Median Peritoneal Cancer Index (PCI) was 16 (I–III quartiles: 8–26) and 25 patients (59%) had at least two PIPAC procedures. Major complications according to the Common Terminology Criteria for Adverse Events (CTCAE; 3 and 4) occurred in three (4%) procedures, and, according to the Clavien–Dindo classification (>3a), one (1%) severe complication occurred. There were no reoperations or deaths within 30 days. Median overall survival (mOS) from diagnosis was 19.6 months (range 14–24), and mOS from first PIPAC was 10.5 months (range 7–13). Excluding cases with very heavy metastatic peritoneal burden, with PCI from 2 to 26, treated with more than one PIPAC, mOS from diagnosis was 22 months (range 14–39). Eleven patients (26%) underwent curative-intent surgery after a bidirectional approach. R0 was achieved in nine (82%) patients and complete pathological response was obtained in three (27%) cases.

**Conclusions:**

Patient selection is associated with bidirectional approach efficacy and feasibility for SPM GC treatment, which may allow potentially curative surgical radicalization in highly selected cases.

Gastric cancer (GC) is responsible for over 1 million new cases in 2020 and an estimated 769,000 deaths, ranking fifth for incidence and fourth for mortality globally.^1^ The peritoneum is a common metastatic site of GC. In a recent population-based study, the incidence of synchronous peritoneal metastases (SPM) was 21%, rising to 40% if we consider studies on patients who underwent staging laparoscopy.^2^

Current standard treatment for GC patients with SPM is palliative systemic therapy, however the prognosis is poor.^3^ These poor systemic chemotherapy results could be explained by the low penetration of chemotherapy agent to the peritoneum layer due to the plasma-peritoneum barrier. Therefore, in the last decades, new chemotherapy delivery techniques were developed to increase local control to peritoneal disease, with promising results.

Currently, hyperthermic intraperitoneal chemotherapy (HIPEC) after cytoreductive surgery (CRS) seems to be the only therapeutic option with curative intent but only in selected patients with low burden peritoneal disease.^4^

A new intraperitoneal chemotherapy delivery technique, pressurized intraperitoneal aerosol chemotherapy (PIPAC), was introduced in 2011, consisting of applying a cytotoxic solution (cisplatin and doxorubicin) nebulized with a micro-pump into the abdominal cavity for 30 min, through laparoscopic access and a normothermic capnoperitoneum with a pressure of 12 mmHg. PIPAC exploits the aerosol solution to allow an homogeneous distribution of drugs within the abdomen. In addition, the capnoperitoneum creates an artificial pressure gradient that overcomes tumor interstitial fluid pressure, which can often represent an obstacle in cancer therapy. The use of PIPAC results in higher local drug concentrations compared with conventional intraperitoneal or intravenous chemotherapy.^5^ At the same time, the plasma concentration of the chemotherapeutic agents remains low, reducing potential adverse effects and toxicity; hence, PIPAC applications can be repeated every 4–6 weeks. For these reasons, this treatment has also been used for peritoneal metastases (PM) of GC in association with systemic chemotherapy, with encouraging results.^6–10^

In February 2019, PIPAC was introduced in the management of patients with PM from GC treated at two dedicated surgical centers in Italy. Cisplatin doses of 7.5 mg/m^2^ body surface in 150 mL NaCl 0.9% followed by doxorubicin at 1.5 mg/m^2^ in 50 mL NaCl 0.9% at 12 mmHg and 37°C for 30 min were used during the first phase of our experiment. Subsequently, drug doses were escalated to 10.5 mg/m^2^ for cisplatin and 2.1 mg/m^2^ for doxorubicin, as suggested by others.^11^

The present manuscript reports the perioperative morbidity and mortality as well as the benefits of using PIPAC in the management of patients with synchronous PM from GC. Our aim was to evaluate the efficacy of PIPAC in combination with systemic chemotherapy as a bidirectional approach for GC patients with PM. Furthermore, the rate of patients undergoing CRS after a bidirectional approach was evaluated.

## Materials and Methods

### Study Design

An observational, retrospective study based on a prospectively maintained database from two high-volume Western surgical centers (Upper GI Surgery Unit of Verona University, and the General and Oncological Surgery Unit of Siena University) was conducted. The data of all patients with SPM from GC who had undergone a bidirectional approach with PIPAC and systemic chemotherapy between October 2019 and April 2022 were included. Pure palliative-intent PIPAC procedures as well as patients with extraperitoneal metastases were excluded. Sex, age at the first PIPAC procedure, systemic chemotherapy regimen used, the extent of PM (determined by the Peritoneal Cancer Index [PCI] according to the Sugarbaker score), ascites volume, length of hospital stay, morbidity and mortality were recorded for each patient. Adverse events were assessed according to the Common Terminology Criteria for Adverse Events (CTCAE) version 5.0^12^ and Clavien–Dindo (CD) classification.^13^ Quantitative variables are reported as median (interquartile range) or median (minimum–maximum) values, while qualitative variables are reported as percentages. Overall survival (OS) was estimated using the Kaplan–Meier method, and the median survival (95% confidence interval [CI]) was stated. The analyses were performed using STATA^®^ version 17.1 statistical software (StataCorp LLC, College Station, TX, USA).

### Surgical Technique

Under general anesthesia, 5 and 10–12 mmHg laparoscopic balloon trocars (Applied Medical, Paris, France) were placed, in accordance with the open laparoscopic technique, and a capnoperitoneum of 12 mmHg at 37°C was applied.

Ascites volume was documented, ascites was removed (sending a sample for cytological examination), an accurate exploratory laparoscopy was performed, and the PCI was calculated. Multiple biopsies were performed in different abdominal quadrants during the first procedure and all following procedures to ascertain the tumor regression grade.^14^

A CAPNOPEN^©^ nebulizer was then connected to an intravenous high-pressure injector and inserted into the upper left side trocar and fixed with a 45° angle to the underlying peritoneum to allow for a better spatial drug distribution pattern and a greater spraying distance between the nozzle head and the underlying small bowel peritoneum compared with that obtained with a perpendicular nozzle position.^15^

The safety protocol with a checklist containing all safety aspects was systematically double-checked before administration of cytotoxic agents. A pressurized aerosol containing chemotherapy agents was then applied.

Initially, a cisplatin dose at 7.5 mg/m^2^ body surface in 150 mL NaCl 0.9% followed by doxorubicin at 1.5 mg/m^2^ in 50 mL NaCl 0.9% at 12 mmHg and 37 °C for 30 min was used (the first 29 procedures). Subsequently, the cisplatin and doxorubicin doses were increased to 10.5 mg/m^2^ and 2.1 mg/m^2^, respectively, as suggested by Tempfer et al.^11^

The system was then kept in steady state for 30 min (application time), and remaining toxic aerosol was exhausted in a closed surgical smoke evacuation system. Finally, trocars were retracted and the laparoscopy ended. No drainage of the abdomen was applied.

## Results

Patients’ clinical and demographic characteristics are described in Table 1. Between October 2019 and April 2022, 74 PIPAC procedures in 42 consecutive patients with Eastern Cooperative Oncology Group (ECOG) performance status (PS) ≤2 were performed. Thirty-two patients were treated in Verona and 10 were treated in Siena. Twenty-seven patients (64%) were female and the median age at first PIPAC was 60.5 years (I–III quartiles: 49–68 years). The median PCI was 16 (I–III quartiles: 8–26). Ascites was detected in 20 procedures (47.6%) from 42 patients at the first PIPAC administration, with a mean volume of 499 mL (minimum–maximum 0–6800 mL). Fifteen of 25 patients had ascites at the second PIPAC administration, with a mean volume of 999 mL (minimum–maximum 0–7800).

**Table 1:** Patients’ clinical and demographic characteristics

Characteristics	n (%)	n (%)
**Patients/PIPAC**	Patients (n = 42)	PIPAC (n = 74)
1 PIPAC procedure	17 (40.4%)	
2 PIPAC procedures	18 (42.9%)	
≥3 PIPAC procedures	7 (16.7%)	
**Drug doses used for PIPAC**		
Cisplatin 7.5 + doxorubicin 1.5	–	29 (39%)
Cisplatin 10.5 + doxorubicin 2.1	–	45 (61%)
Age, years	60.5 (49–68)^a^	
*Sex*		
Male	15 (35.7%)	
Female	27 (64.3%)	
CRS ± HIPEC	11 (26%)	
PCI	16 (8–26)^a^	
*Ascites*		
First PIPAC		20; 499 mL (0–6800)^b^
Second PIPAC		15;999 mL (0–7800)^b^
Postoperative stay		5 (±2)^c^
*Organ resection*		
Adnexectomy	7 (17%)	
*Major complications*		
CTCAE (III, IV)		2 (2%)
Clavien Dindo >3a		0
*Mortality*		
30-day mortality	0	
90-day mortality	7 (17%)	

*PIPAC* pressurized intraperitoneal aerosol chemotherapy, *CRS* cytoreductive surgery, *HIPEC* hyperthermic intraperitoneal chemotherapy, *PCI* Peritoneal Cancer Index, *min* minimum, *max* maximum, *CTCAE* Common Terminology Criteria for Adverse Events

^a^Median (p25–p75)

^b^n; median (min–max)

^c^Mean (SD)

In seven procedures, bilateral ovariectomy for Krukenberg lesions was performed before PIPAC in the same operation, as ovaries were the only site of disease progression at computed tomography (CT) scan, and the lesions were also symptomatic. In the present series, most patients received systemic chemotherapy before and after PIPAC.

**Fig. 1:**
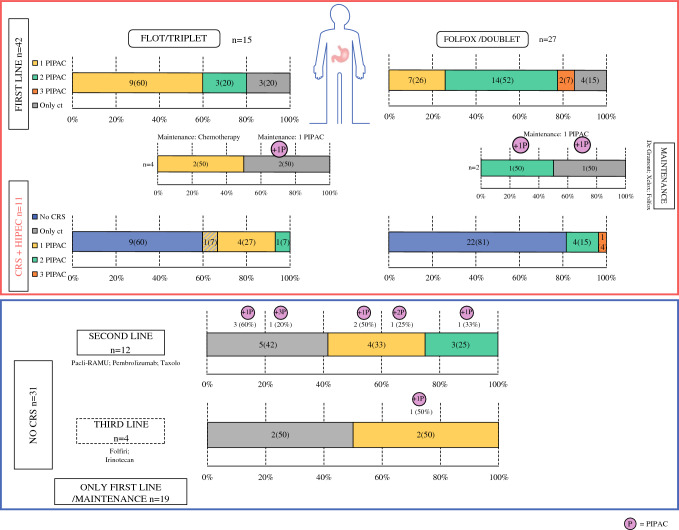
Temporal relationship between PIPAC and systemic chemotherapy, and details of the therapeutic schemes administered for each patient. *PIPAC* pressurized intraperitoneal aerosol chemotherapy, *CRS* cytoreductive surgery, *HIPEC* hyperthermic intraperitoneal chemotherapy

As this was a retrospective study, there was heterogeneity in treatment schedules and in the number of PIPAC procedures performed and the timing of PIPAC procedures compared with systemic chemotherapy. Figure 1 summarizes the bidirectional therapy approach; each patient was coded according to the number of PIPAC procedures performed during first-line treatment, and this code was then maintained throughout the oncological pathway.

It should be noted that after first-line treatment with FOLFOX/other doublet or FLOT/other triplets, a non-negligible percentage of patients (26%) underwent CRS and HIPEC. Most frequently, resection with curative intent was performed after a first-line triplet schedule. Patients who did not proceed to surgery after first-line therapy and who continued with a second/third line received at least two PIPAC procedures, even during the chemotherapy regimens for advanced disease (Fig. 1).

Twenty-five patients (59%) had at least two PIPAC procedures: the Peritoneal Regression Grading Score (PRGS) after two PIPAC procedures was ‘1’ in three patients, ‘2’ in 13 patients, and ‘3’ in nine patients; in patients who underwent three PIPAC procedures, the last PRGS was ‘3’ in four patients, one patient had a PRGS value of 2, and in one patient the PRGS was 1.

According to CTCAE 3 criteria, major complications occurred in two (2%) procedures, while according to the CD classification (>3a), any severe complication occurred. Furthermore, two patients (2%) had CTCAE 3, CD 2 events, including one lipothymia and blood loss in one patient that required transfusion; this was also the only major surgery-related complication.

Regarding minor complications, five patients had a CTCAE 1, CD 1 event (6%): two had a fever, one had subcutaneous emphysema associated with trocar insertion, one had vomiting that was treated with an antiemetic, and one had hyperbilirubinemia; two patients had a recurrence of ascites (CTCAE 2, CD 3a).

There were no reoperations or deaths during the hospitalization and within 30 days of surgery. The mean hospital stay was 5 days (±2), with only two 30-day readmissions. Seven patients (17%) died before 90 days from the last PIPAC, mainly due to disease progression and not to events related to the intraperitoneal procedure.

**Fig. 2:**
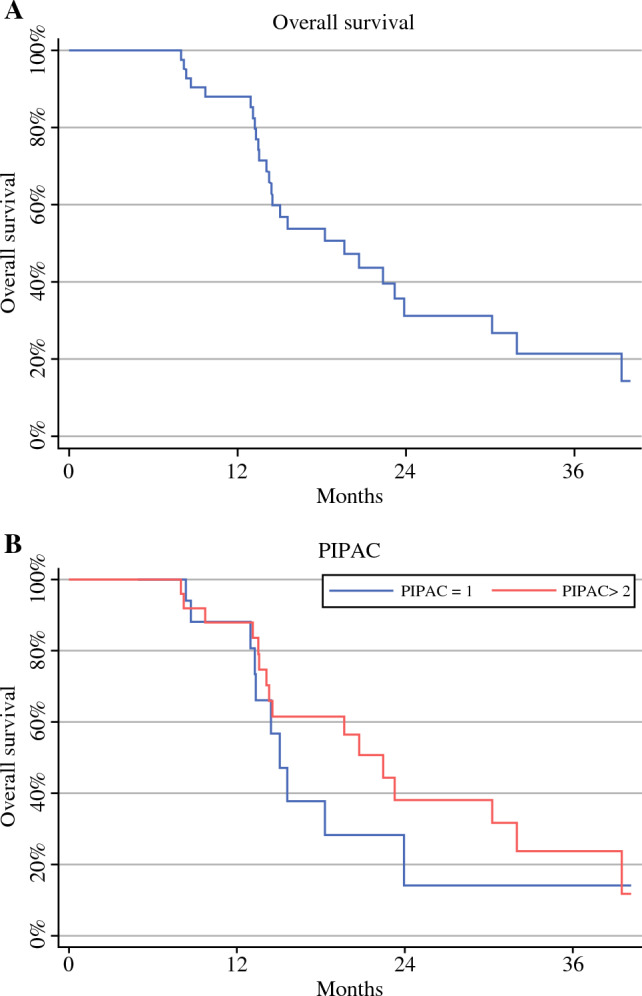
Median overall survival from diagnosis in the whole series (**A**) and according to the number of PIPAC procedures [one vs. two or more] (**B**). *PIPAC* pressurized intraperitoneal aerosol chemotherapy

**Fig. 3:**
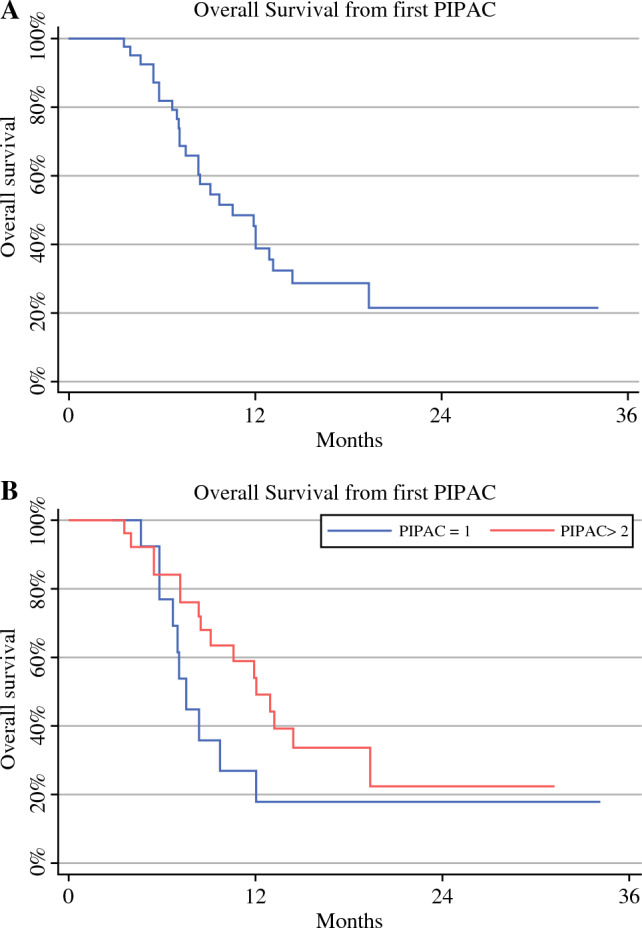
Median overall survival from first PIPAC in the entire series (**A**) and according to the number of PIPAC procedures [one vs. two or more] (**B**). *PIPAC* pressurized intraperitoneal aerosol chemotherapy

**Fig. 4:**
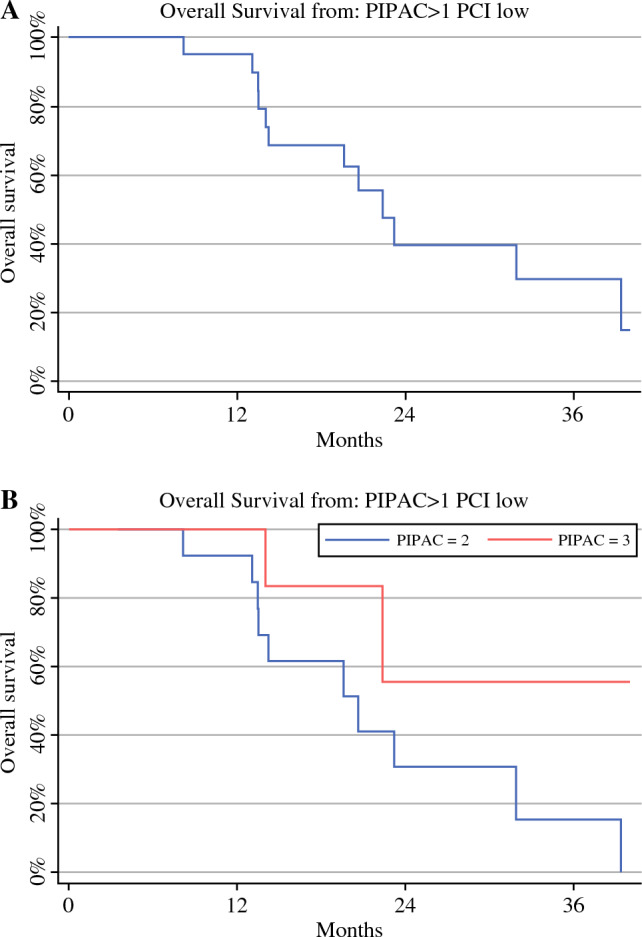
Median overall survival from diagnosis in the selected series, i.e. *n* = 20 patients with limited PCI (between 2 and 26) who were treated with more than one PIPAC (**A**) and according to the number of PIPAC procedures [two vs. three] (**B**). *PIPAC* pressurized intraperitoneal aerosol chemotherapy, *PCI* Peritoneal Cancer Index

**Fig. 5:**
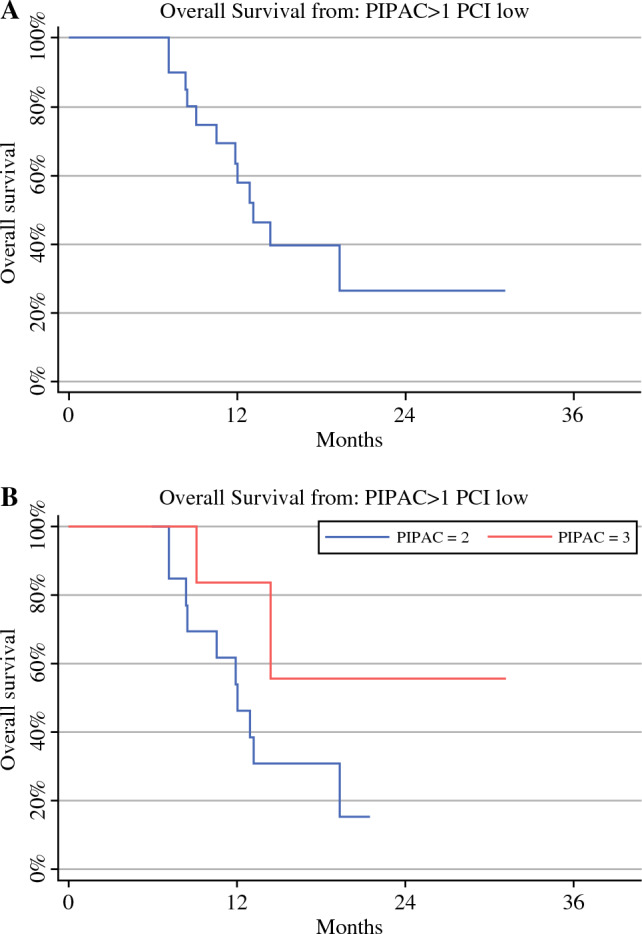
Median overall survival from first PIPAC in the selected series, i.e. *n* = 20 patients with limited PCI (between 2 and 26) who were treated with more than one PIPAC (**A**) and according to the number of PIPAC procedures [two vs. three] (**B**). *PIPAC* pressurized intraperitoneal aerosol chemotherapy, *PCI* Peritoneal Cancer Index

In the entire series, the median OS (mOS) from diagnosis was 19.6 months (range 14–24). mOS from diagnosis was 15 months (range 13–24) in patients who underwent one PIPAC, and in cases treated with two or more PIPAC procedures, the mOS was 22 months (range 14–32) (Fig. 2a, b). Also in the entire series, the mOS from the first PIPAC was 10.5 months (range 7–13). mOS from the first PIPAC was 7.5 months (range 6–12) in patients who underwent one PIPAC and 12 months (range 8–19) in cases treated with two or more PIPAC procedures (Fig. 3a, b). Of note, after excluding cases with heavy metastatic peritoneal burden and considering only the 20 patients with more limited PCI (from 2 to 26) who were treated with more than one PIPAC (two PIPAC procedures in 13 cases and three PIPAC procedures in seven patients), a mOS from diagnosis of 22 months (range 14–39) was reported. In subjects who underwent two PIPAC procedures, the mOS from diagnosis was 21 months (range 13–32), and in cases who underwent three PIPAC procedures, the median was not reached (Figs. 4a, b). In this selected series of 20 patients, the mOS from the first PIPAC was 13 months. mOS from the first PIPAC was 12 months (range 8–19) in patients who underwent two PIPAC procedures, and in cases treated with three PIPAC procedures, the median was not reached (Fig. 5a, b).

Eleven patients (26%) in the entire series underwent curative-intent surgery after a bidirectional approach. Ten cases underwent total gastrectomy with D2 lymphadenectomy and peritonectomy in the areas affected by the disease at the time of diagnosis. In one case, an Ivor–Lewis esophagectomy was performed. A HIPEC procedure was added in seven cases, while multivisceral resection was performed in five cases: one patient received transverse colon resection and bilateral salpingo-ovariectomy, one patient received right flexure and transverse colon resection plus jejunal resection, one case underwent splenectomy and left adrenalectomy, and two cases underwent bilateral salpingo-ovariectomy (Table 2). In patients who underwent curative-intent surgery, the systemic chemotherapy regimen was FOLFOX in five cases, while FLOT/DOX were chosen in six cases; the number of PIPAC procedures before going for radical gastrectomy was three in one patient, two in five patients, and one in six cases.

**Table 2:** Clinicopathologic characteristics of patients who underwent CRS ± HIPEC

Age	Histology	Chemo	PCI	PIPAC (*n*)	Type of surgery	HIPEC	R status	pTNM	TRG	Complications (CD)	Status	Survival (days)
65	Signet	FLOT	8	1	GT	Yes	R0	ypT0N0M0	1	I	Alive	881
54	Signet	FOLFOX	3	2	GT + colic and jejunal resection	Yes	R0	ypT4aN3M1	5	IIIa	Death	250
49	Signet	FOLFOX	4	2	GT +colic resection, bilateral ovariectomy	Yes	R1	ypT4aN3M1	5	IIIa	Death	457
67	Signet	FLOT	4	1	GT	No	R0	ypT1bN3M1	3	IIIa	Death	232
57	Signet	FOLFOX	2	3	GT	No	R0	ypT3N0M0	2	–	Alive	696
43	Signet	FOLFOX	26	2	GT + bilateral ovariectomy	Yes	R0	ypT2N0M1	3	II	Death	916
64	Mucinous	FOLFOX	6	2	GT	Yes	R0	ypT4N3M0	3	I	Alive	365
48	Mixed	DOX	10	2	GT + bilateral ovariectomy	Yes	R0	ypT0N0M0	1	II	Alive	963
56	Tubular	FLOT	12	1	Ivor–Lewis esophagectomy	No	R0	ypT0N0M0	1	IIIa	Death	554
63	Signet	FLOT	6	1	GT	Yes	R1	ypT4aN1M1	2	II	Alive	719
71	Tubular	DOX	3	1	GT + spleen and left adrenal gland resection	Yes	R0	ypT4aN1M0	3	IIIa	Alive	326

*CRS* cytoreductive surgery, *HIPEC* hyperthermic intraperitoneal chemotherapy, *Chemo* chemotherapy, *PCI* Peritoneal Cancer Index, *PIPAC* pressurized intraperitoneal aerosol chemotherapy, *TRG* tumor regression grade, *CD* Clavien–Dindo, *FLOT* fluorouracil, leucovorin, oxaliplatin and docetaxel, *FOLFOX* folinic acid, fluorouracil and oxaliplatin, *DOX* doxorubicin

The number of postoperative complications, according to the CD classification, was two in CD 1 (18%; one polyuria, one chylous ascites), three in CD 2 (27%; one urinary tract infection, one pleural effusion, one acute renal failure), and five in CD 3a (45%; three thoracenteses, one esophago-jejunal fistula, one pneumothorax). No major complications (CD >3a) or postoperative mortality occurred.

R0 was achieved in nine (82%) patients operated on with radical intent. In two cases, R1 resection was obtained. Of note, macroscopic residual disease (R2) was not present in any patient and complete pathological response, both at the primary site and the metastatic level, was obtained in three (27%) cases.

## Discussion

Treatment of PM from GC is an emergent problem. In the last years, there has been an increase in the incidence of GC (cardia and non-cardia GCs combined) among young adults aged <50 years in both low- and high-risk countries.^1^

Over the last decades, in Western countries, tumors with Laurèn intestinal histology located in the distal third of the stomach have decreased in favor of locally advanced proximal and Laurèn diffuse-type tumors with a higher risk of peritoneal dissemination.^16^ Therefore, it is clear that young patients with PM are increasing in clinical practice, and managing these patients is becoming a great challenge for surgical oncologists.

Currently, the standard treatment for GC patients with PM is systemic chemotherapy, with a mOS of 8–13 months.^3,17–19^ The last Italian Research Group for Gastric Cancer guidelines (GIRCG) stated that some patients with unresectable stage IV GC could benefit from combined treatment with a systemic treatment followed by radical surgery.^20^

Regarding stage IV patients with peritoneal involvement, local intraperitoneal chemotherapy seems to improve survival in addition to surgery. The CYTO-CHIP study^4^ showed that in selected patients with PM from GC, HIPEC after CRS offers better outcomes than surgery alone, in terms of both survival and recurrence rate. These advantages were shown irrespective of the tumor histology for both the poorly cohesive carcinoma (PCC) and non-PCC groups (OS rates at 1, 3, and 5 years were 76.3, 48.3, and 38.6%, respectively, in the CRS/HIPEC group versus 54.5, 22.0, and 18.4%, respectively, in the CRS-only group). However, the benefits were more evident for patients in the non-PCC group with better OS than the PCC group. Moreover, the gain in survival is also related to the PCI status of patients: patients in the PCC group with a PCI ≤ 6 and in the non-PCC group with a PCI ≤ 12 have the best benefits from HIPEC after radical surgery. Therefore, the best treatment for selected patients with low-burden peritoneal disease currently consists of systemic chemotherapy followed by radical surgery and HIPEC.

However, the question regarding treatment of the ‘intraperitoneal side’ is still open, both for possible enhancement in the setting of limited disease addressed to CRS and HIPEC and for patients who have a higher PCI (> 6 in the PCC group and > 12 in the non-PCC group) who could still benefit from a bidirectional approach combining local intraperitoneal treatment with systemic chemotherapy.

In this setting, a new intraperitoneal chemotherapy delivery technique, know as PIPAC, was developed in 2011. This technique applies a cytotoxic solution (cisplatin and doxorubicin) nebulized with a micro-pump into the abdominal cavity for 30 min, through laparoscopic access and a normothermic capnoperitoneum with a pressure of 12 mmHg. The technique significantly improved intraperitoneal drug delivery, patient outcome, and survival with preserved quality of life, and has been used for PM of various origins, with encouraging results in GC.^6–8^

The low toxicity and surgical impact of this new procedure allows for use in combination with systemic chemotherapy in a bidirectional setting without increasing drug toxicity.^7–10^

Based on the above, in 2019 this new intraperitoneal chemotherapy delivery technique was introduced in clinical practice at two high-volumes centers in Verona and Siena for patients with PM from GC, in association with systemic chemotherapy. Initially, the only aim was to reduce ascites-related symptoms. Considering the efficacy and safety of the treatment confirmed by several studies,^7–10^ we expanded the indication to treat patients without ascites and with a lower PCI.

In the present study, we aimed to report the safety and efficacy of the bidirectional approach in our series of 74 PIPAC procedures in 42 patients, all with SPM from GC without extraperitoneal disease.

In our series, the bidirectional approach was considered safe; major complications according to CTCAE 3 occurred in 2% of patients, while no major complications, according to the CD classification (>3a), were reported. In the current literature, a recent systemic review^21^ found that major adverse events of CTCAE 3 ranged from 0.7 to 25% of procedures, while those of CTCAE 4 ranged from 0 to 4.1%. CD >3a was reported up to 11.8%.^21^

The lower complication rate in our series could be explained by the exclusion of patients with ECOG PS ≥3 who received PIPAC only with pure palliative intent. Indeed, in our prior experience, in the purely palliative setting, we realized that patient selection is essential to avoid extreme complications, as confirmed by others.^9,10^

The absence of 30-day mortality in the present series mainly reflects the good short-term results described. On the other hand, the 90-day period (i.e. 17%) is not related to complications but to the inclusion of patients with advanced disease, or high PCI or ascites >4 L, or even in cases who underwent three PIPAC procedures and who therefore died within 90 days of the last treatment but after a long oncological history.

The main findings of the present study are the global mOS of 19.6 months (range 14–24) and the impressive median survival of 22 months (range 14–39) that were achieved when we selected cases who underwent a true bidirectional approach, i.e. at least two PIPAC procedures, without heavy peritoneal spread (PCI >26). Another strength of the present study is the high rate (26%) of patients who underwent CRS ± HIPEC.

In the literature, other reports are available but in almost all the other studies, the number of PIPAC procedures performed in a bidirectional context was relatively low compared with our series. Moreover, the other studies also included patients with metachronous peritoneal carcinomatosis and/or extraperitoneal disease.

Furthermore, Nadiraze et al.^6^ reported a retrospective series of 60 PIPAC procedures in 24 patients, but only eight PIPAC procedures were performed in a bidirectional scheme; four patients also had extraperitoneal metastases and the mOS was 15.4 months. Khomyakov et al.^7^ published a prospective study of 56 PIPAC procedures in 31 patients, only seven of whom had synchronous PM, with a median of one bidirectional PIPAC; a mOS of 13 months was reported.

More recently, two studies^9,10^ analyzed PIPAC in a bidirectional approach for gastric PM. Alyami et al.^22^ described a series of 163 PIPAC procedures in 42 patients without extraperitoneal disease, reporting an OS of 19.1 months; metachronous PM cases were also included and the rate of CRS was 14.3%. Di Giorgio et al.^10^ published a series of 46 PIPAC procedures in 28 patients (only 12 had SPMs and six also had extraperitoneal disease); the mOS was 15 months, the median number of PIPAC procedures was 1.7, and only one CRS + HIPEC was reported.

The study by Tidadini et al.^23^ was the only comparative non-randomized study of systemic chemotherapy only (29 patients) and bidirectional PIPAC + chemotherapy (17 patients) in patients with PM, both synchronous and metachronous, without extraperitoneal disease. The mOS was 9.1 months in the systemic chemotherapy-only group and 12.8 months in patients treated with the bidirectional approach.

In our series, the mOS of 19.6 months was higher when compared with almost all other reports, and similar to that reported by Alyami et al.,^22^ mainly due to the clear inclusion criteria that we choose by excluding patients with extraperitoneal disease and metachronous metastases. Indeed, to our knowledge, the present series is the largest series on the specific clinical setting of synchronous PM from GC without distant metastases, and thus may represent a good piece of evidence to define the benefits of this new treatment option.

Moreover, when we selected cases who underwent a true bidirectional approach, i.e. at least two PIPAC procedures, in patients with PCI from 2 to 26, an impressive median survival of 22 months (range 14–39) was achieved.

Some studies support a relationship between the number of PIPAC procedures and OS.^10,24–26^ The mOS reported by Di Giorgio was 9 months for those who received one PIPAC compared with 15 months for those who underwent more than one PIPAC. Sindayigaya et al.^24^ reported a mOS of 9 months with none to two PIPAC procedures, compared with 16 months for three or more PIPAC procedures (*p *= 0.0003). Similarly, De Simone et al.^25^ reported a mOS of 18 months in patients who underwent at least two PIPAC procedures. Finally, Gockel et al.^26^ also reported a significant difference in mOS based on the number of PIPAC procedures received, increasing from 121 days for the group who underwent one to two PIPAC procedures, to 450 days for those who underwent three or more PIPAC procedures (*p *= 0.0376). In part, these findings are related to selection bias consisting of having treated younger patients with more PIPAC procedures, with better general conditions, and likely with more limited disease. However, at least partly, the improved survival observed with more procedures is related to the effectiveness of proper bidirectional treatment.

The higher rate of patients who underwent CRS, even compared with the series by Alyami et al.^22^ (26% vs. 14.3%), with acceptable postoperative morbidity and no mortality but with a high rate of R0 resections and some complete pathological responders in our series, opens the horizons for a combined multimodal approach including systemic, intraperitoneal treatment and radical surgery with curative potential.

Based on this evidence, the Upper GastroIntestinal Surgery of Verona, Italy, has promoted a multicenter clinical trial, the PIPAC VerONE trial,^27^ focused on patients with GC with limited PM, whose primary objective is to compare the R0 resection rate in patients treated with systemic chemotherapy alone or with both chemotherapy and PIPAC. The Italian Medicines Agency (AIFA) approved the study design in October 2021 and recruitment is currently ongoing (trial registration: EUDRACT 2021-000830-33; NCT 05303714).

## Conclusion

Our results suggest that proper patient selection is associated with high feasibility and efficacy of the bidirectional approach, including PIPAC plus systemic chemotherapy in the treatment of synchronous peritoneal carcinosis from GC, which, in some cases, may also allow an attempt at surgical radicalization and cure in the context of this emergent and often lethal clinical problem.
